# Exotropia in a Patient With a Novel Homozygous 4-Hydroxyphenylpyruvate Dioxygenase-Like Protein (HPDL) Variant

**DOI:** 10.7759/cureus.83070

**Published:** 2025-04-27

**Authors:** Yuka Kasuya, Tadahiro Mitani, Kumiko Noguchi, Teru Mizuno, Shinji Makino, Hitoshi Osaka

**Affiliations:** 1 Ophthalmology, Jichi Medical University, Shimotsuke, JPN; 2 Pediatrics, Jichi Medical University, Shimotsuke, JPN

**Keywords:** 4-hydroxyphenylpyruvate dioxygenase-like gene, exotropia, progressive spastic paraplegia, retinal discoloration, strabismus

## Abstract

The 4-hydroxyphenylpyruvate dioxygenase-like protein (*HPDL*) is a mitochondria-localized protein involved in the biosynthesis of coenzyme Q10 in the electron transfer system, and its variants have been reported to cause progressive neurodegenerative diseases such as neonatal leukoencephalopathy and hereditary spastic paraplegia.

In this case report, we present a case of a nine-year-old girl with exotropia with a novel *HPDL* variant who underwent strabismus surgery. She was referred to the ophthalmology department with exotropia and a history of progressive spastic paraplegia with gait disturbance. Brain MRI showed no remarkable findings. Whole-exome sequencing revealed a homozygous variant c.1040delC (p.Thr347Metfs*66) in the *HPDL* gene. Ophthalmic examination revealed a best-corrected visual acuity of 20/12 in both eyes. Fundoscopy showed retinal discoloration at the level of the retinal pigment epithelium in the right eye. As the patient had intermittent exotropia with good convergence, she was followed up conservatively. One year after the initial examination, the patient could not keep her eyes in a central position by convergence. The alternate prism cover test revealed exotropia of 80 prism diopters. We diagnosed that intermittent exotropia had deteriorated into constant exotropia. The patient’s family requested a strabismus surgery, which was conducted under general anesthesia. Standard left lateral rectus recession, left medial rectus resection, and right lateral rectus recession were also performed. Postoperatively, her exotropia was reduced, and she achieved good convergence. The patient and her family were satisfied with the surgical outcome, and no recurrence was noted one year postoperatively.

Our results provide important information for the associations of variant in *HPDL* with progressive spastic paraplegia, strabismus and retinal changes and broaden the genetic spectrum of HPDL-related disease. This is the first report to present a novel *HPDL* variant and document the performance of strabismus surgery for constant exotropia.

## Introduction

In 2020, Husain et al. [1] reported the various clinical presentations of the 4-hydroxyphenylpyruvate dioxygenase-like protein (*HPDL*) variant, from severe cases with acute encephalopathy-like symptoms in the neonatal period, extensive white matter lesions in the cerebral white matter, Leigh encephalopathy-like findings to mild cases of spastic paraplegia in the teenage years with no obvious lesions on brain magnetic resonance imaging (MRI).

Coenzyme Q10, an endogenously synthesized lipid molecule, is known for its role as a cofactor within the mitochondrial respiratory chain, where it functions in electron transfer and ATP synthesis. In 2021, Banh et al. [2] reported that 4-hydroxymandelate, an intermediate of coenzyme Q10 biosynthesis in the mitochondrial electron transfer system, is a product of HPDL. Thus, HPDL is a substrate of coenzyme Q10.

To date, >90 cases of HPDL variants have been reported in 59 families [3]. Although it has been noted to cause visual impairment and strabismus, no detailed descriptions of strabismus exist.

We present the case of a nine-year-old girl with exotropia with a novel *HPDL* variant who underwent strabismus surgery.

## Case presentation

**Figure 1:**
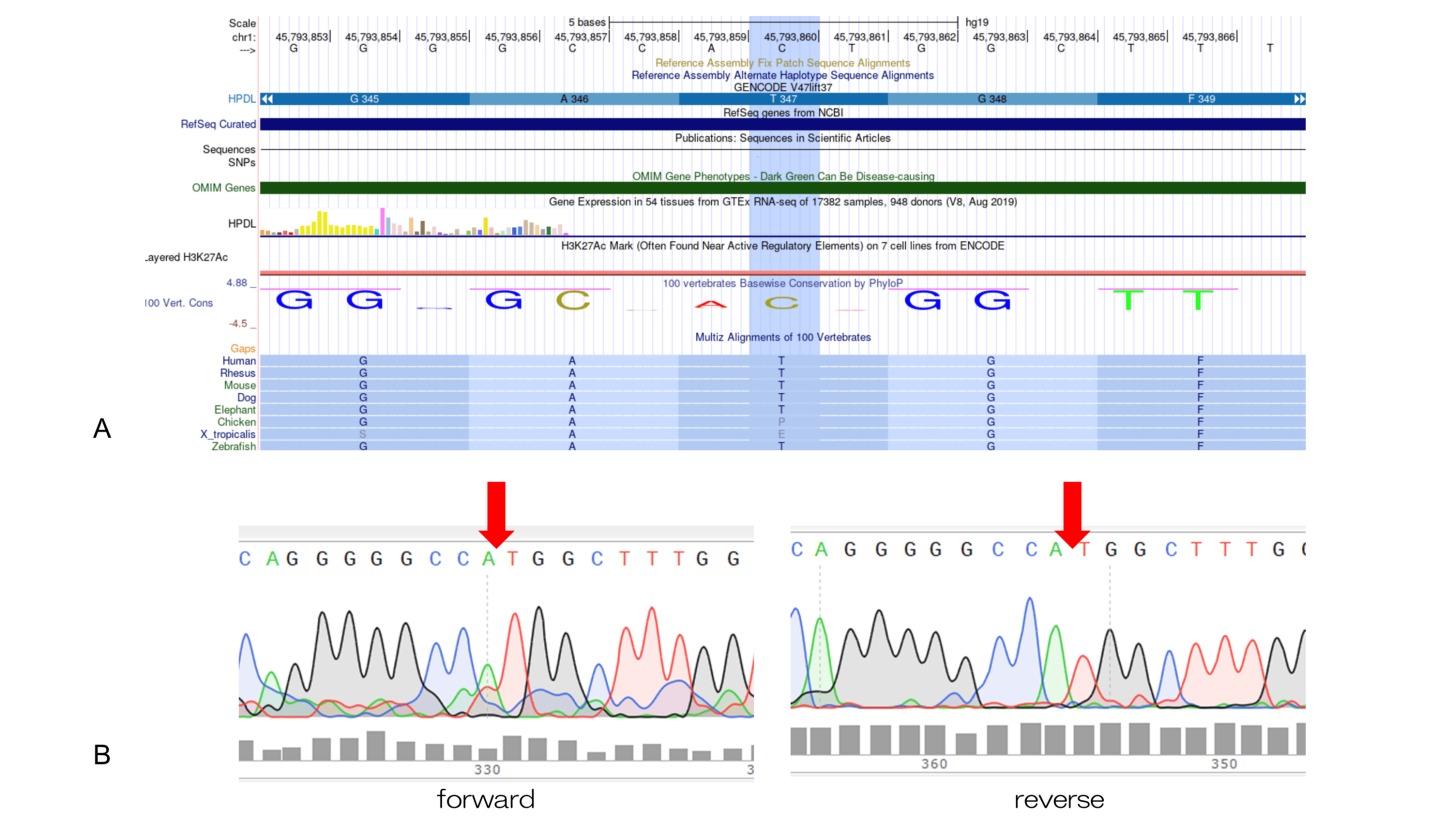
Genetic analyses (A) Analysis results using the University of California Santa Cruz (UCSC) genome browser. (B) Sanger sequencing was used to validate the homozygous variants of HPDL in this patient. Red arrows indicate the variant (c.1040delC).

A nine-year-old Japanese girl was referred to the ophthalmology department with exotropia in her left eye. She had a history of progressive spastic paraplegia with gait disturbance that developed when she was seven years old. Brain MRI showed no remarkable findings. Whole-exome sequencing revealed a homozygous variant c.1040delC (p.Thr347Metfs*66) in the HPDL gene (NM_032756.4), and these candidate mutations were validated by Sanger sequencing (Figures 1A, 1B).

**Figure 2:**
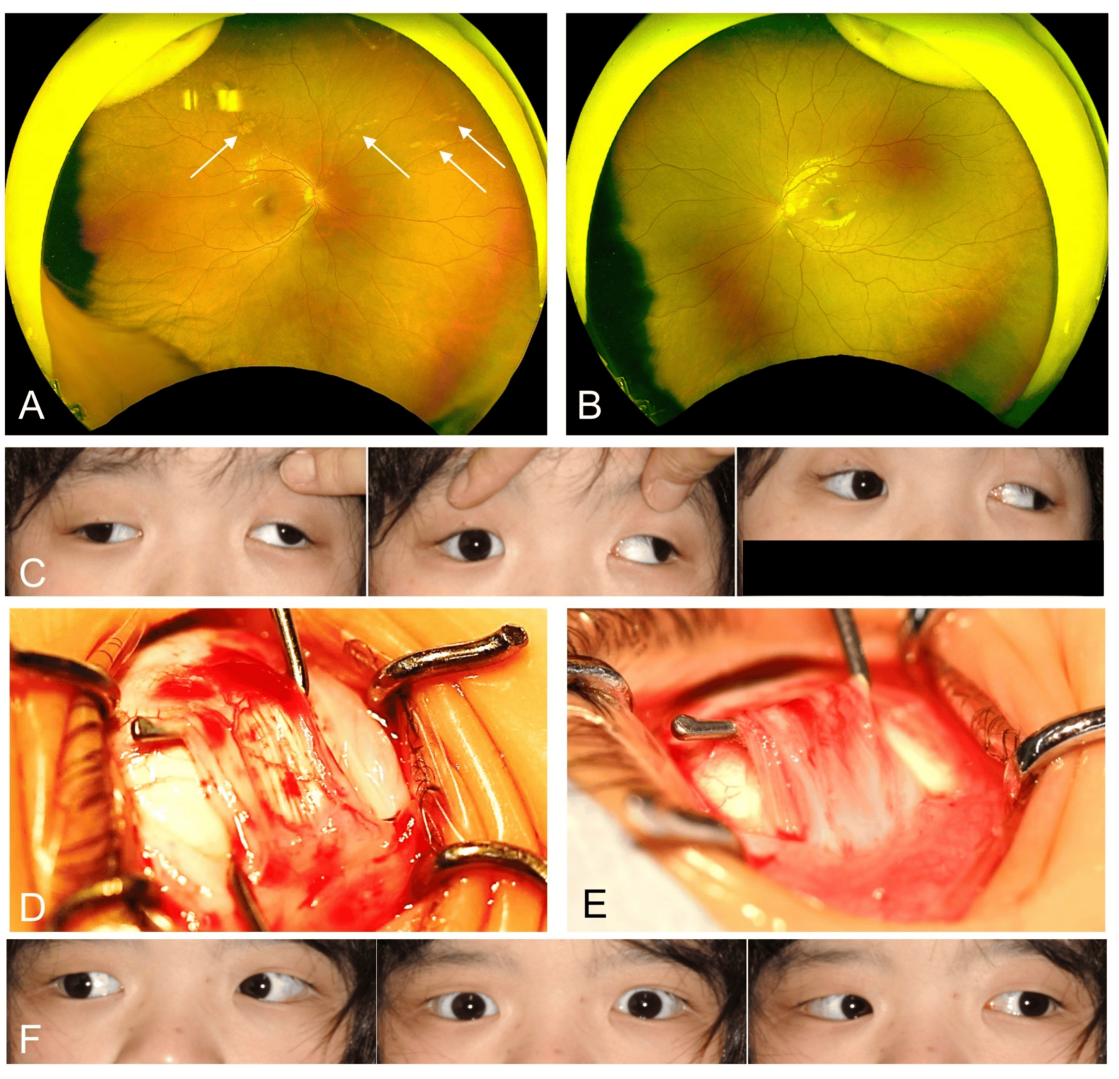
Ophthalmological findings Fundus photographs of the right (A) and left (B) eyes. Note retinal discoloration at the level of the retinal pigment epithelium in the right eye (arrows in A). (C) Preoperative ocular motility photographs in the horizontal gaze position demonstrate large exodeviation in the left eye. Surgical photographs of the left lateral (D) and medial (E) muscles show no atrophy. (F) Postoperative ocular motility photographs in the horizontal gaze position depict improvement in exodeviation.

The initial ophthalmic examination revealed a best-corrected visual acuity of 20/12 -1.75 diopters [D] in the right eye and 20/12 -2.25D in the left eye. Slit-lamp examination did not demonstrate any other abnormalities in either eye. Fundus photographs of the right and left eyes are shown in Figures 2A, 2B. Fundoscopy showed retinal discoloration at the level of the retinal pigment epithelium in the right eye (Figure 2A). As the patient had intermittent exotropia with good convergence, she was followed up conservatively.

One year after the initial examination, the patient could not keep her eyes in a central position by convergence. The alternate prism cover test (APCT) revealed exotropia of 80 prism diopters (PD) in her left eye. We diagnosed that intermittent exotropia had deteriorated into constant exotropia. The patient’s family requested a strabismus surgery, which was conducted under general anesthesia. Photographs of preoperative ocular motility in the horizontal gaze position are shown in Figure 2C. Standard left lateral rectus recession (9 mm), left medial rectus resection (10 mm), and right lateral rectus recession (9 mm) were also performed, and no thinning of the lateral or medial rectus muscles was observed (Figures 2D, 2E). Postoperatively, her exotropia was reduced; the APCT showed 10 PD and 8 PD left exotropia and hypertropia of her left eye, respectively (Figure 2F), and she achieved good convergence. The patient and her family were satisfied with the surgical outcome, and no recurrence was noted one year postoperatively. Surgical correction of eye misalignment is effective in the acquisition of binocular function.

## Discussion

To our knowledge, this is the first case to present this novel *HPDL* variant and document the performance of strabismus surgery on a nine-year-old girl with this *HPDL* variant for constant exotropia.

Regarding the ophthalmological findings of *HPDL* variants, nine cases (52.9%) were noted to have visual impairment and strabismus was noted in 17 participants [1], and among 31 participants, 11 cases (35.5%) had ocular motility disorder [4]. However, no detailed descriptions of strabismus have been provided, and no reports on strabismus surgery and fundus findings in *HPDL* variants have been published. The exact incidence of strabismus associated with *HPDL* variants is not clear, and its mechanism is unknown. Rather than the strabismus angle, the clinical problem is whether the convergence is possible or not, i.e., whether it is intermittent or constant exotropia. The fact that convergence became possible in this case postoperatively indicates that functional improvement was achieved. On the other hand, we speculated that in this case discoloration at the retinal pigment epithelium could be a polar bear track [5], which may be a coincidental association.

Herein, whole-exome sequencing identified the homozygous variant c.1040delC (p.Thr347Metfs*66) in the *HPDL* gene, and these candidate mutations were validated by Sanger sequencing in the present case. According to the interpretation of the American College of Medical Genetics and Genomics (ACMG) criteria, this novel frameshift was classified as “uncertain significance (PM2_ Supporting)”. However, this mutation is predicted to be “possibly damaging” with a score of 0.904 (sensitivity: 0.82; specificity: 0.94) in silico analysis (Polyphen-2). We believe that *HPDL* is likely to be the pathogenic gene due to its extremely rare frameshift variant and phenotypic similarity to phenotypes previously reported in *HPDL*-related diseases. Because it is a novel *HPDL* variant, it is not exactly clear how this frameshift was involved in strabismus and retinal changes. This frameshift leads to the formation of abnormal proteins that must be investigated to determine whether they are degraded or alter the function of HPDL. Currently, we are conducting a histological study of the extraocular muscles at surgery and of coenzyme Q10 activity in skin fibroblasts.

## Conclusions

In the present case, initial intermittent exotropia progressed to constant exotropia, and eye position abnormality was speculated to worsen. Furthermore, we speculated that discoloration at the retinal pigment epithelium could be a polar bear track, which may be a coincidental association.

Our results provide important information for the associations of variant in *HPDL* with progressive spastic paraplegia, strabismus and retinal changes and broaden the genetic spectrum of *HPDL*-related disease. Further ophthalmological findings in cases with *HPDL* gene variants are necessary to elucidate the pathogenesis of strabismus.
